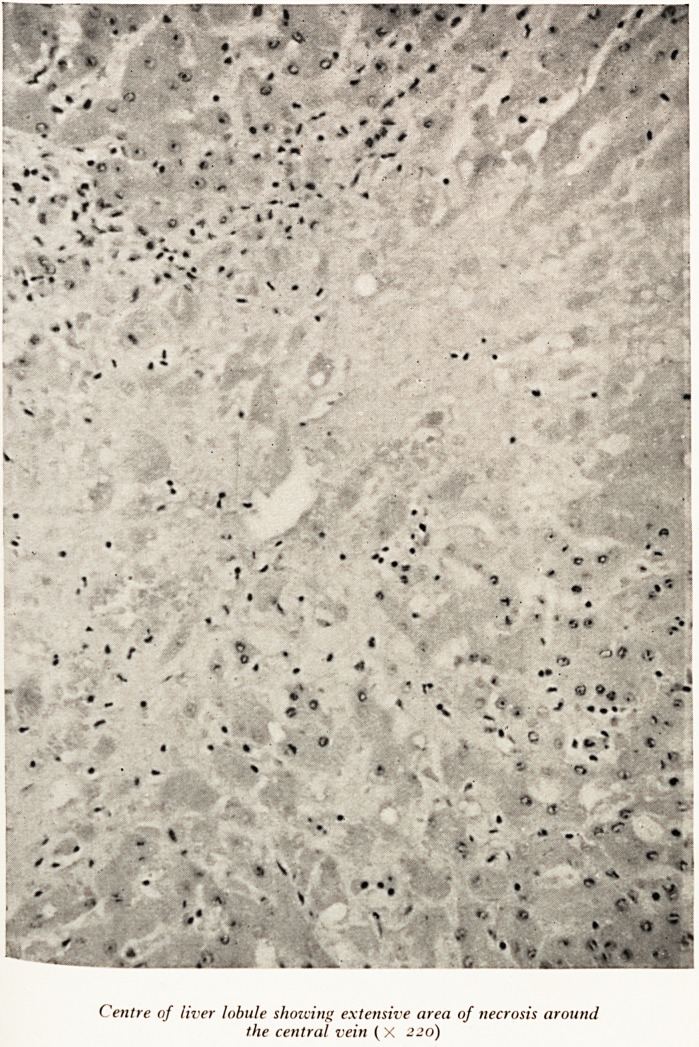# Case Report—Jaundice with Hepatic Failure

**Published:** 1955-09

**Authors:** T. F. Hewer


### JAUNDICE WITH HEPATIC FAILURE—A CASE ILLUSTRATING DIAGNOSTIC PROBLEMS

### A Clinico-Pathological Conference of the University of Bristol Medical School

CHAIRMAN: PROFESSOR T. F. HEWER

*Professor Hewer:* The woman we are discussing this evening was first attended by Dr. Eastman, her general practitioner.

*Dr. F. V. Eastman:* This lady was one of the well-known patients—always at the surgery! Until this illness started it had always been a question of " nerves ". It was not until September 1953 that she began to have symptoms of organic disease. She complained her heart was beating fast and of some upper abdominal discomfort. She managed to get over this and I did not see her again until the end of the month, when she was jaundiced. There was no story of running a high temperature. She was in the Bristol Royal Infirmary within a fortnight and four weeks later when she left hospital she was happy and bright. Her jaundice was less but was still present. I was called to see her again early in the New Year and then she was not so well. The previous afternoon she had vomited, she had abdominal distension and I thought she had an intestinal obstruction. I sent her to hospital straight away and was sorry to learn that she died three or four days later, on January 7th, 1954.

*Mr. R. V. Cooke:* This is a very instructive case and I am sure my medical colleagues will support me when I say that these cases are difficult to diagnose. I remember this problem very well because we spent a long time discussing it on one of the ward rounds. She had been jaundiced for five weeks at least when I saw her first. That is not a very long time as I have known a case go on for three months, even longer, before finally clearing up—without surgery! However, we went into the story very carefully as one can learn much from the history. There was in the history a definite suggestion of the onset of hepatitis. She had not been well before the jaundice became evident and, looking at the chart, there was still a little fever—enough to suggest an infective process. The absence of pain did not mean anything conclusive. When she was in hospital in October she was very jaundiced: it would need to be a severe infective hepatitis to bring about so much jaundice. Surgical jaundice can occasionally be painless even if it is due to a stone. The liver was palpable two-fingers breadth below the costal margin. Should we proceed to laparotomy or should we resort to various liver function tests? Rightly or wrongly I felt there was not time to wait for a result of such tests; and they might well have been unhelpful because the pathologist should be given a case early if a definite answer is to be given. I should like to hear which of the tests in these circumstances are regarded as valuable and how much value is placed on them. I think they are of very little value unless done early. The surgeons are equally confident and they are often wrong! A cholecystogram was done but even that was not a very great help. The opaque material failed to show up the gall-bladder: that perhaps rather tended to suggest a surgical gall-bladder and I decided we would explore her (on October 28th, 1953). We found the gall-bladder was not enlarged. This was a shrunken gall-bladder with a stone in it and I removed it. There might have been other stones in the common duct but it was not thickened. There was nothing to feel in it and nothing to justify exploring it: no enlargement, no thickening of its wall and normal bile on aspiration. Even if there is no enlargement and nothing to feel, you still may find, on opening the common duct, a stone or stones. Even when you have opened the common duct you may fail to find a stone. We could find nothing on exploration but even then I was not too confident that I had emptied the duct of any stone. There was no pancreatic lesion. There was a group of enlarged glands at the liver hilum: I took one out and we learnt nothing from it—there were " simple reactive changes " only. Her recovery from the operation was uninterrupted. She was getting better from her original condition but not because of anything we did to her! She went out of hospital an unsolved problem except that we had excluded any surgical obstruction, so we had not operated unnecessarily. We thought perhaps she was one of those long-standing cases of jaundice due to primary liver disease and that it might possibly clear up.

When this woman came into hospital again (January 2nd, 1954) she was very ill and dying and we could do nothing.

*Dr. R. L. Bishton* asked Mr. Cooke if he was averse to doing a liver biopsy. Mr. Cooke replied that the liver was very swollen and he was afraid of causing haemorrhage; having done a useless operation, except to exclude anything surgical, he decided not to do a biopsy.

*Professor T. F. Hewer:* The liver was very swollen?

*Mr. R. V. Cooke:* It was terribly swollen and looked the liver of an obstructive jaundice; but it must have been a hepatitis.

*Professor Hewer* asked Dr. J. Guy to tell them something about liver-function tests.

*Dr. J. Guy:* I think I can agree with Mr. Cooke. I can remember this lady. We had at the time considered whether it was worth doing a test; she had been jaundiced for four to six weeks and the chances of getting an answer were very scanty.

In the early stages of jaundice a distinction can often be made between obstructive and hepatocellular jaundice by the thymol turbidity or flocculation tests, which give higher figures in the latter. In the absence of bone disease, a serum alkaline phosphatase of over 40 units is almost diagnostic of obstructive jaundice, and one of under 15 units of hepatocellular jaundice. In this case the results would probably have been ambiguous.

*Dr. O. C. Lloyd* (describing the autopsy): This patient was jaundiced. The other external abnormality was the operation wound which had been healing very nicely and was still a purple colour. There was more than a litre of yellow fluid in the abdomen. There were well-marked vascular anastomoses between portal and systemic systems including the adhesions which had been formed by the wound. Blood vessels all stood out in a characteristic pattern and these findings of fluid in the abdomen and dilatation of anastomoses showed that during the course of these three months there had been developing some degree of portal hypertension. The liver was very much smaller than expected and this is interesting in view of the observation at operation that it was enlarged. There was slight roughening of the surface and nodular irregularity. The spleen was much congested and enlarged. The congestion was partly recent and partly chronic. It was a passive venous congestion. (Plate XIX.) The portal hypertension had not been going on long enough to produce fibrosis of spleen. The gall-bladder was missing. The bile-ducts were not dilated or obstructed in any way and there was no evidence of obstruction in the past. The liver showed the changes of early cirrhosis, such as are found after two or three months of an acute hepatitis that has not cleared up. The bile ductules (Plate XX) seemed to be increased in numbers, but this may have been not so much evidence of proliferation, as the result of atrophy of the parenchyma which supported them. Bile thrombi were seen. (Plate XXI). All the cells around the central vein had died. This liver cell death was not the original necrosis when she first had her attack of acute hepatitis. We know from the experience of those who have done serial biopsies on livers, that the necrotic remains of the cells get removed completely in about five or six weeks. This suggested to me that there had been some acute liver cell death within the last four or five days. The reason for this was probably poor blood flow in the central parts of the lobules of the liver.

Here is the case of a woman who had acute hepatitis, whose liver was observed to be enlarged after five weeks, whose jaundice did not clear up, who went on to develop a mild degree of portal cirrhosis and who, in consequence of this portal cirrhosis,

PLATE XIX

*Liver and spleen*

PLATE XX

*Periphery of liver lobule showing some small bile-ducts dilated with bile* (× *220*)

PLATE XXI

*Centre of liver lobule showing extensive area of necrosis around the central vein* (× *220*)

developed portal hypertension. I think there is good evidence in this case that the cause of death was hepatic failure.

*Mr. R. V. Cooke:* Is it just the liver failure that kills them?

*Dr. Lloyd:* I think it was one of the important causes in her. In acute hepatitis there can be far more necrosis yet they seem to recover because the liver parenchyma has a wonderful power of regeneration.

*Professor Hewer:* Even in this case, with so much survival of the parenchyma, death was due to hepatic failure. The function of the liver cells must have been seriously impaired by the ischaemia that so recently produced the centrilobular necrosis. Even the regenerated liver cells must have been suffering from severe ischaemia and could not perform their proper function.

*A Student:* Was a reticulin stain done?

*Dr. Lloyd:* No, it was not. A reticulin stain gives you a very good idea of the liver lobule architecture. But I did a van Giesen stain which shows the portal fibrosis equally well and also gives distinction between the old colagen fibres and the new ones formed as a result of more recent disease.

*Professor Hewer:* In addition to the diagnostic interest of this case there is the further, most important, observation that the liver shrank from a state of enlargement, actually observed at operation, to a small cirrhotic organ in only eight weeks. This is in keeping with what we know of some other cases of hepatitis that run an unfavourable course and give rise to portal cirrhosis.

## Figures and Tables

**Figure f1:**
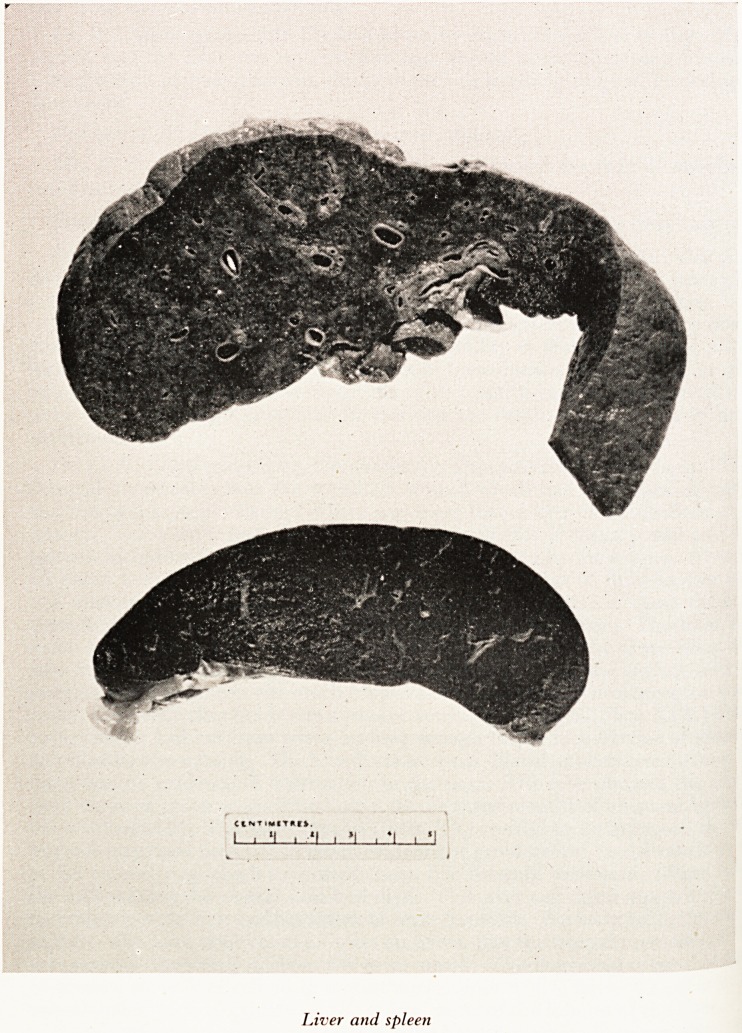


**Figure f2:**
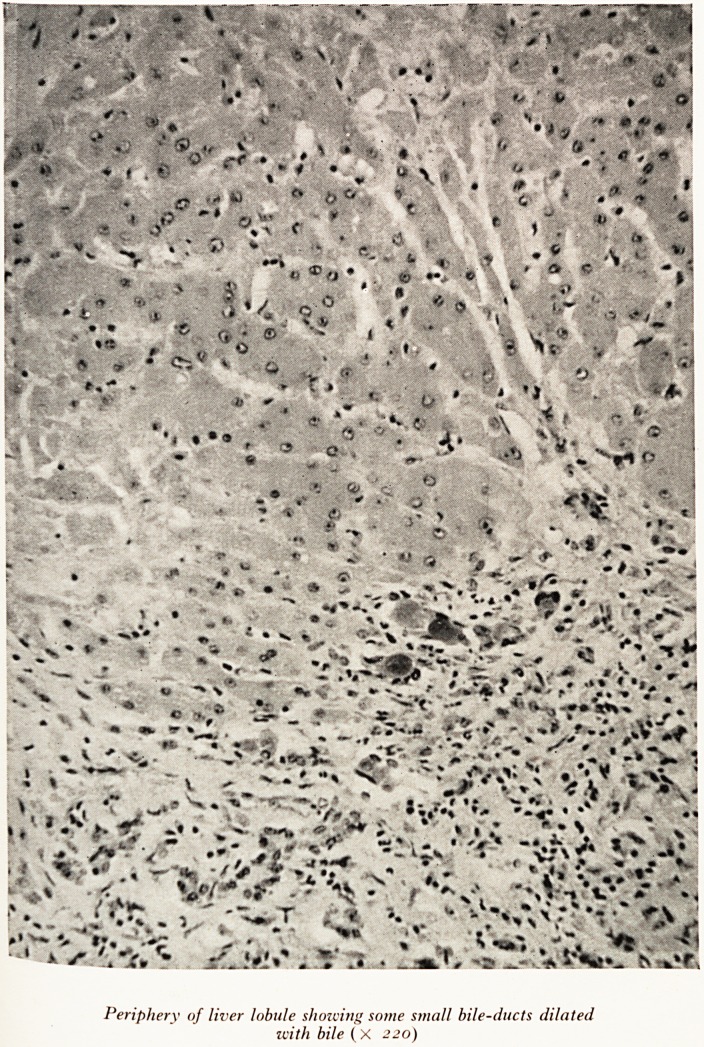


**Figure f3:**